# Enhancing Safety for Separating Families Affected by Domestic and Family Violence: A Scoping Review of Modifiable Factors

**DOI:** 10.1177/15248380251325195

**Published:** 2025-03-20

**Authors:** Kristel A. Krella, Felicity L. Painter, Anna T. Booth, Amy Holtzworth-Munroe, Elizabeth Evans, Heng Jiang, Jennifer E. McIntosh

**Affiliations:** 1 La Trobe University, Melbourne, VIC, Australia; 2 Indiana University, Bloomington, USA; 3 Federal Circuit and Family Court of Australia, Melbourne, VIC, Australia

**Keywords:** domestic and family violence, safety, separation, family law, legal system

## Abstract

Relationship separation constitutes a period of significant risk for families, with many parents and children experiencing an escalation in domestic and family violence (DFV) as they move through the separation process. While research demonstrating associated risks and adverse impacts is well documented, modifiable factors associated with increased safety for this vulnerable group have received less attention. This scoping review addresses this gap. Informed by the Preferred Reporting Items for Systematic Reviews and Meta-Analyses extension for Scoping reviews (PRISMA-ScR), articles were retrieved from the Medline, PsycINFO, CINAHL, Embase, and SCOPUS databases. Results were limited to peer-reviewed articles reporting original empirical data, in English. No date restrictions were applied. In the resulting 17 eligible studies, we found inconsistent definition and measurement of safety underscored by a paucity of research focusing on safety as a primary outcome for separating families affected by DFV. Of the available evidence, socioecological factors associated with enhanced safety during this period included physical distance from the perpetrator, strategic use of technology to enhance safety, relational support, survivor-centered service support, and intentional processes to aide safety within the family law and court systems. At the individual level, parents’ active role in navigating safety for themselves and their children, particularly in the face of systemic and service barriers, appears key. Consideration is given to the interface of systems, socioecological and individual factors that may collectively promote safety from DFV during the family separation process.

Domestic and family violence (DFV) is a national and international public health and human rights concern (World Health Organization [WHO], 2021). DFV refers to violent, threatening, or other behavior of one family member (including current and former intimate partners) toward another that coerces or controls them, or causes them to be fearful (Neilsen, 2011). This includes any physical violence, sexual violence, stalking, or psychological harm (Breiding et al., 2015). Epidemiological research demonstrates the disproportionate vulnerability of women and children in this context (Tjaden & Thoennes, 2000), with serious and long-lasting impacts well documented (Dillon et al., 2013; Kalmakis & Chandler, 2015; McIntosh, 2003; Noble-Carr et al., 2020). In line with this, multilevel factors (i.e., individual, relational, community, and societal) associated with increased risk of DFV are well established (Capaldi et al., 2012; Stith et al., 2004). However, evidence regarding factors associated with increased safety for families affected by DFV is reportedly less common (Gerino et al., 2018) and often extrapolated from risk-orientated research through the absence of risk factors, leading to inferences about safety that are theoretical and untested. This review is focused specifically on DFV in the context of relationship separation (i.e., between former intimate partners), where children are involved.

While it is important to understand risk and to predict the likelihood of violence reoccurring (as is the focus of most risk assessment approaches; Spiranovic et al., 2021), it is equally important to understand factors that increase safety and protection for women and children who have experienced DFV. This is in line with the national principles of Australia’s National Research Organization for Women’s Safety (ANROWS) and the framework of World Health Organization for preventing violence against women, which call for the incorporation of protective factors in evidence-based risk assessment (Toivonen & Backhouse, 2018) and prioritization of women’s safety (UN Women & Social Development Direct, 2020).

This scoping review seeks to map and identify gaps in the existing literature regarding safety for an important subpopulation disproportionately affected by DFV—separating families. Involvement with the family law system further exacerbates risk for separating families, and targeting risk factors (e.g., substance use, mental health) or perpetrator behavior alone is insufficient, as it does not speak directly to the experience of safety for survivors. As such, safety in this review is conceptualized as a subjective report of feeling safer. Importantly, the focus is on identification of *modifiable* factors associated with safety, as unlike non-modifiable factors (e.g., age, gender, genetic factors, race, ethnicity), these can be influenced by processes and interventions aimed at enhancing safety.

## Separated Versus Intact Family Contexts

While it has been reported that physical violence declines after relationship separation, research demonstrates that the controlling behaviors of perpetrators often continue (Hayes, 2012), and risk of lethality for women and children escalates (Campbell et al., 2003). In their concept analysis of post-separation abuse, Spearman et al. (2023) identified multiple ways that women and children continue to experience abuse through deliberate patterns of intimidation by the perpetrator, including legal and economic abuse, threats and endangerment to children, isolation, harassment, and stalking. In this way, factors associated with the re-establishment of safety for this group are likely to differ from intact families. This is consistent with research demonstrating changes in safety strategy use across time and context (Haselschwerdt et al., 2016).

## The Family Law Context

The legal systems and policies that regulate divorce, separation, and parenting arrangements are a key context influencing the ability to achieve and maintain safety following separation from an abusive partner (Jaffe et al., 2008). However, the adversarial family law context itself can create conditions for abusive behaviors to continue or emerge following separation. An Australian House of Representatives Standing Committee on Social Policy and Legal Affairs inquiry (2003) into parenting arrangements following separation received a vast amount of evidence regarding the animosity that adversarial legal proceedings can create, when parties are pitted against each other in a climate of preexisting tension and conflict. The enquiry found focus was often shifted away from reaching agreements in children’s best interests, toward *winning* or using the system to exact revenge for hurt caused by the other party.

The term *legal abuse* (or *systems abuse*; Douglas & Ehler, 2022) has been used to describe the abusive parents’ use of court processes to further exert coercive control (Gutowski & Goodman, 2023), for example, by intentionally prolonging court proceedings (Douglas, 2018), seeking child contact arrangements for the purpose of exerting power (Elizabeth, 2017), distorting information to make the protective parent appear unfit (Elizabeth, 2017), or financially burdening the other parent with the costs of protracted litigation (Miller & Smolter, 2011). Legal abuse can lead to, or exacerbate, psychological distress, anxiety and depression, post-traumatic stress symptoms, economic instability and poverty, and a loss of faith in the legal system (Douglas, 2018; Fitch & Easteal, 2017; Gutowski & Goodman, 2023). Enduring violence between caregivers is also a contributor to intergenerational disadvantage, with children experiencing serious and pervasive impacts that follow them into adulthood. This includes the disruption of neurological and biochemical pathways during crucial stages of development, contributing to a range of adverse physical and mental health outcomes (McIntosh, 2003).

Where DFV is not correctly identified at entry to the legal system (e.g., mistaken for mutual conflict), or undisclosed by survivors (e.g., due to safety reasons), abusive behaviors of the perpetrator may go undetected (Feresin et al., 2018), resulting in proceedings and outcomes that place children and their protective parent at risk of further harm (Francia et al., 2020; Khaw et al., 2021; Morrison, 2015; Shorey & Baladram, 2024). Such processes and outcomes may include the protective parent being forced to endure cross-examination by the perpetrator during proceedings, or the child being ordered to have unsupervised visits with the abusive parent. Research has shown that evidence of DFV may also be dismissed or minimized by the courts, even when it is disclosed by survivors or correctly identified (Hester, 2011). As Hester (2011) demonstrates in her Three Planet Model of approaches to women’s and children’s safety, family law systems may order contact between abusive caregivers and children, even if this is in direct contradiction with the assessments, safety plans, or orders of the DFV, criminal justice, and child protection systems.

## Approaches to the Study of Enhanced Safety

### Protective Strategies, Help-Seeking, and Their Effectiveness

Consistent with Bronfenbrenner and Morris’ (2007) bioecological systems perspective, intentional processes and actions that enhance safety can be instigated at the individual, family, community, and service levels. At the individual level, the active engagement of survivors in creating or finding safety for themselves through the use of a range of strategies is increasingly well documented, marking a shift from historic perspectives of survivors as passive victims without agency (Goodman et al., 2003). Hamby (2013) defines *protective strategies* as any action survivors take to minimize the likelihood of experiencing risk or loss. According to Hamby (2013), these strategies tend to fall into two categories: private and public. Private strategies are used in isolation, and include placating (e.g., doing as the perpetrator wants), resisting (e.g., trying to avoid the perpetrator), and safety planning (e.g., creating an escape plan; Goodman et al., 2003). Existing research in this space has typically involved intact families, and findings have produced mixed results, with some strategies (e.g., resisting and placating) shown to *increase risk* for survivors, or to decrease in effectiveness over time, or in certain contexts (Nixon et al., 2017; Parker & Gielen, 2014; Renner & Hartley, 2022). This highlights the importance of safety plans and strategies that are individualized and safety-oriented versus those purely risk averse (Parker et al., 2015).

Public strategies link the individual with another individual or service in their micro- or exosystem (Bronfenbrenner & Morris, 2007), through disclosure and *help-seeking* interactions with social support networks and public agencies, including informal (e.g., family, friends, neighbors), formal (e.g., DFV services, counselors), and legal (e.g., police, lawyer; Goodman et al., 2003) services. Concerningly, little attention has been given to the efficacy of interventions that occur following help-seeking, including which elements of the intervention or agency are most associated with intended outcomes (Hamby et al., 2015). For example, Vikander et al. (2023) analyzed mothers’ strategies to safeguard themselves and their children post-separation. While highlighting important ways that women interact with society’s protective interventions (e.g., DFV services, child protection, police, family law), circumstances under which these interactions led to increased safety and protection for themselves and their children were not elaborated. This is an important gap as research has shown that interventions or factors that are intended to improve safety (e.g., being granted an intervention order [IVO] or access to services) do not necessarily translate to actual safety or increased feelings of safety for all women and children (Nixon et al., 2017).

In the gray literature too, DFV program evaluation reports are plentiful, but fraught with barriers in evaluating effectiveness (e.g., lack of funding to devote to these efforts and limited skills of staff in conducting evaluations; Sullivan, 2011). This is layered with complexities such as the unique needs and circumstances of each survivor, debate regarding which outcomes or measures are appropriate, anonymity and short-term nature of many services, and the potential to compromise safety through the process, leading to some services resisting evaluation efforts (Sullivan, 2011).

### Perpetrator Intervention

In line with calls for perpetrator accountability in the DFV sector, focus has increased on perpetrator intervention as a means to reduce violence and consequently increase survivor safety. The effectiveness of such interventions in preventing recidivistic violence, and hence increasing safety, has been subject to criticism (Travers et al., 2021). Of the limited evidence examining their effectiveness, there are numerous concerns regarding the outcomes evaluated and measures used (A. Day at al., 2019). For example, the perception of program *completion* as the primary outcome of behavior-change programs provides no evidence of changed risk status, nor its translation to the home and family context, particularly improved safety for the survivor, and the intentional actions and behaviors that support this. The salience of survivor-centered and safety-focused approaches in research is, to date, under-represented.

## The Study of Safety During Separation

Given separating families experiencing DFV are a significantly vulnerable population requiring particular attention, knowledge of factors associated with the promotion or re-establishment of safety appears key. To this end, a scoping review was conducted to address the question: “What factors are associated with enhanced safety in separating families affected by domestic and family violence?” In light of the complex and dynamic nature of *separation* in the context of DFV, separating and separated are used interchangeably to include families that have physically separated, and may be engaged with a legal process regarding their separation.

## Methods

### Design

Due to the varied contexts, perspectives, and disciplines characterizing DFV enquiries, a scoping approach was taken to map and synthesize the available evidence regarding modifiable factors associated with safety for separating families affected by DFV and to identify gaps in knowledge (Tricco et al., 2018). This review was undertaken using the Preferred Reporting Items for Systematic Reviews and Meta-Analyses extension for Scoping Reviews (PRISMA-ScR) protocol and followed the PRISMA-ScR checklist to improve the quality and accountability (Tricco et al., 2018).

### Eligibility Criteria

Inclusion criteria were peer-reviewed published studies reporting on empirical data, in English, with safety as either a primary outcome or a secondary finding, in legally separating or physically separated families (e.g., engaged in a family law process, current IVO, or residing in a DFV shelter) where DFV has been alleged. No date restrictions were applied. Studies with overlapping samples were not excluded. Studies were excluded if they focused on increased risk only and interpreted the inverse to equate to increased safety, focused on a proxy for safety (e.g., coping, resilience, improved mental health symptomology, or certain court/mediation outcomes alone), or did not report findings for an identifiable sample of separated/separating families (e.g., reported findings were not disaggregated from intact families).

### Information Sources and Search

The search was conducted in September 2023 using five research databases: SCOPUS, PsycINFO, CINAHL, Embase, and Medline. The database search strategy was developed, piloted, and refined in consultation with a specialist health-science librarian. The search was guided by the PCC framework (population, concept, and context) for scoping reviews (Pollock et al., 2023) and was constructed in line with four key concepts: population (caregivers/children), exposure (DFV), context (relationship separation), and outcome (safety).

Our definition of safety focused on a subjective report of felt safety. Given limited results, the key terms within the concept for safety were expanded and truncated in order to capture all terms with the same root word. Key terms within the remaining concepts were also expanded, including MeSH terms and truncated words. A full search strategy (including search terms and their combinations) for PsycINFO is detailed in Table 1. The search strategy was used with all databases following necessary adjustments to MeSH terms, truncations, wildcards, and Boolean operators. A snowballing approach was used to identify any additional papers by scanning the reference lists of included records.

**Table 1.** PsycINFO Search Strategy.

Key terms			
Concept 1: Context	Concept 2: Population	Concept 3: Exposure	Concept 4: Outcome
“Family court” “Law system” “Legal system” “Family Law” Separat* Divorce Mediation “Dispute resolution” Divorce** Mediation**	Caregiver Carer Grandparent Mother Father Youth Child* Parent* Victim-survivor Victim Survivor “Young people” Caregivers** Grandparents** Parents**	“Family Violence” “Domestic Violence” “Family and Domestic Violence” “Intimate Partner Violence” “Domestic abuse” “Domestic and family violence” “Partner abuse” “Spousal abuse” Domestic violence** Intimate partner violence**	Safe* Secur* “Decreas* risk” “Diminish* risk” Victimi#ation Protect*

** Indicates MeSH term.

### Selection of Sources of Evidence

The search identified 2,410 records. Three additional studies were identified using snowballing techniques. Following the removal of 1,332 duplicates, 1,081 records were screened at title and abstract level using Covidence (www.covidence.org), a web-based systematic review software platform, designed to facilitate the independent selection process and aid in the resolution of disagreement in consensus. To ensure consistency, three reviewers screened the titles and abstracts of the same 20 publications and discussed the results, informing the development of a screening tool (see Appendix). Interrater reliability was adequate (>70%) to continue with the screening process without any revisions to the tool. Based on these criteria, the lead author screened all titles and abstracts, and an additional two reviewers screened 20% each. Studies that did not meet the inclusion criteria were excluded. Those focused on risk were put through to full text to examine whether safety later emerged as a secondary finding. Conflicts were resolved as they arose via conferencing. One reviewer screened all papers at the full-text level. Of these, all papers that met the inclusion criteria were double screened by a second reviewer. Any that were labeled “maybe” were decided upon via conferencing between two independent reviewers. Of the remaining 254 records, 237 were excluded at the full-text level. These records were excluded for three main reasons. Firstly, they did not focus on safety as a primary outcome (e.g., they focused on adverse impacts and risk factors associated with DFV, or a proxy for safety). Secondly, they were not the correct study design (e.g., they were a review). Lastly, they were the wrong population (i.e., they did not report findings for a separated/separating subsample because they did not focus on this population or they mixed findings with families that were intact). This distinction is important as research demonstrates that strategies differ for separating families (Haselschwerdt et al., 2016). Consistent with the objectives of the review to map and synthesize the available evidence, and identify gaps in knowledge, articles were not excluded based on quality. This is consistent with PRISMA-ScR guidelines.

### Data Charting and Items

An extraction table was jointly developed by three reviewers to determine which information and variables to extract. The lead author extracted the study characteristics and findings of each study and inputted them into the table (See Table 2). The three reviewers discussed the results and continuously updated the extraction table in an iterative process. All three reviewers confirmed accuracy of the data extracted. Data were extracted on article characteristics (e.g., country of origin), aim, study design, sample/context, predictor variable (if applicable), safety outcome, and relevant findings.

**Table 2.** Characteristics of Included Studies and Summary of Findings.

Author (Year), Country	Aim	Study Design, Data Collection Intervals	Sample (*N*; % Female), Context, Participant Characteristics (Where Reported: Parent Status, Racial Identification/Ethnicity, SES); Attrition	Predictor/Baseline Variable(s) and Measurement	Safety Outcome(s) and Measurement	Finding(s)/Effect Size(s)
Boethius et al. (2023), Sweden	To explore the experiences of women who had experienced DFV from a former partner, including their interactions with social networks and decision to file a police report	Qualitative (Interviews)	Swedish women (*N* = 21) recruited from a help center for abused women (aged 27–55, further characteristics NR). Some had children (demographics NR)	N/A	Subjective report of protection via interview	Survivors used technology to increase protection through finding support and information; monitoring the perpetrator; storing evidence; and keeping in touch with friends and family. In contrast, perpetrators used technology to harass and/or monitor the movements of survivors in conjunction with other forms of abuse
Bomsta and Sullivan (2018), USA	To examine mothers’ perceptions of how receipt of flexible funding and brief advocacy designed to increase their housing stability may have also impacted their children’s safety, stress, mood, and behavior	Qualitative (Interviews, Longitudinal) T1 (30 days), T2 (3 months), T3 (6 months)	Women (*n* = 42, *M*_age_ = 34) who received a flexible funding intervention through a housing and DFV service; 79% African American; 7% multiracial, 5% African immigrant, 2% white/Caucasian, 7% did not specify; all had children; (*n* = 86, *M*_age_ = 2); attrition at T3: 14%	A brief intervention of flexible funding and brief advocacy	Subjective report via interview	Women reported increased safety and security for them and their children as a result of flexible funding (used for rental arrears, security deposits, moving expenses etc.) and housing stability. The women reported that it provided safety from the perpetrator (e.g., by moving to an undisclosed location), and the dangers of homelessness (e.g., exposure to environmental risk). This finding persisted after 6 months
Booth et al. (2023), Australia	To examine if screening increases safety and the utility of the FL-DOORS as a repeated measure for detecting change in safety and wellbeing over time, in the context of mediation	Quantitative (Longitudinal), T1 (Intake), T2 (~8 weeks post-intake)	Clients (*N* = 67; 64%; M_age_ = 38.1) attending an Australian mediation service; 94% metropolitan service, 6% regional/rural service.	Compulsory completion of pre-mediation risk screening: FL-DOORS (McIntosh & Ralfs, 2012)	Post-mediation risk screening: FL-DOORS (McIntosh & Ralfs, 2012)	The entire sample reported reduced concern for child safety at T2 (*M* = 0.52, *SD* = 0.96) compared to T1 (*M* = 1.38, *SD* = 1.36); lower self-safety concern at T2 (*M* = 0.79, *SD* = 1.39) than T1 (*M* = 2.16, *SD* = 2.11) and lower concern for behaving unsafely at T2 (*M* = 0.15, *SD* = 0.79) than T1 (*M* = 0.60, *SD* = 1.07). All T1–T2 time effects were significant
Clough et al. (2014), USA	To explore DFV survivors’ experiences accessing affordable, safe, and stable housing	Qualitative (Interviews)	Women (*N* = 11, *M*_age_ = 32.8) who had experienced DFV and were accessing housing and DFV programs after leaving an abusive relationship; all had children; 45% white; 36% Latina; 18% African American; 18% employed; income $15,000; 46% were college educated	N/A	Subjective report via interview	Women reported the use of effective strategies to maintain a safe environment for them and their children in the face of individual and systemic barriers regarding housing, for example, staying with friends and family due to limited housing options offered by services. They also identified the importance of trained, compassionate, and persistent practitioners at housing and DFV agencies to achieve safety and stability
Dragiewicz et al. (2021), Australia	To investigate women’s experiences of TFCC	Qualitative (Interviews)	Australian female survivors (*N* = 20, *M*_age_ = 39.0) of TFCC recruited from local women’s legal support programs and health services. 45% Australian, 5% Aboriginal, 50% born overseas	N/A	Subjective report of safety via interview	Technology played a key role in women’s capacity to predict, survive, and manage men’s violence, especially in the absence of adequate social, legal, and structural responses to violence. Behaviors included abstaining from technology use, or enduring electronic surveillance to avoid escalation of threats
Fotheringham et al. (2013), Canada	To evaluate a project designed to enhance the physical, emotional, and psychological safety of children exposed to DFV and high conflict custody and access disputes	Mixed methods**	Canadian families (*N* = 15; 37 individuals) engaged in custody/access disputes and participating in the SFT program. 21% father, 32% mother, 1% step-parent, 46% child (aged 10–19; *M*_age_ = 14)	SFT program	Subjective report of safety via interview; file analysis	*Child report*: Increased safety through safe and supported interactions with their lawyer, and therapist *Parent report*: Confirmed support for children’s involvement and view that children felt safe to express feelings *File analysis*: Showed children’s safety led to increased disclosure of abuse, in turn leading to legal decisions that positively impacted their safety by reducing the possibility of ongoing trauma
Fox et al. (2018), USA	To identify the needs of American Indian women in a DFV shelter in Arizona	Qualitative (Interviews using survey)	American Indian women (*N* = 37, *M*_age_ = 37.0) who were invited to participate in a survey on their move-out date of a DFV shelter; 59% had children	DFV service provider	Subjective report via survey of having safety needs met by service provider	All parents reported that either “all” or “some” of their safety needs (e.g., safety planning, learning about DFV/options, leaving violent relationship) were met by the service (83.8%, 16.2%, respectively); 58.3% reported that “all,” 13.9% reported that “some” of their children’s safety needs were met by the service (27.8% did not want/need this). Key factors/mechanisms of change reported by staff included working collaboratively with clients (guided by their unique circumstances), and provision of immediate longer term (3 years) accommodation
Hauge and Kiamanesh (2020), Norway	To explore how immigrant women experience and negotiate their everyday life with children prior to and after leaving a violent partner, including their encounters with assistance services after exposure to DFV	Qualitative (Interviews)	Immigrant women (*N* = 23) staying at domestic violence shelters with their children; 82% immigrant, 22% Norwegian; SES varied	N/A	Subjective report of DFV services providing women with practical and economic support that led to protection for them and their children	Women normalized their new living arrangements by creating and maintaining predictable routines for their children to protect them and establish a sense of stability. Most women required economic, practical, and emotional support from services to establish these conditions, for example, support with finding accommodation, financial aid, and getting their children into day care/school
Holtzworth-Munroe, Applegate, et al. (2021), USA	To report 1-year follow-up outcomes from a randomized controlled trial comparing traditional litigation with two mediation approaches designed to protect parent safety	Quantitative (Longitudinal), T1 (Initial), T2 (1-year)	T1: As reported below T2: Traditional litigation (*n* = 68); shuttle mediation (*n* = 53); videoconferencing mediation (*n* = 58)	Case management pathway (traditional litigation, shuttle mediation, videoconferencing mediation)	(1) Follow-up interview completed by parents, questions derived from standardized measures including the MASIC (past year DFV scores, Holtzworth-Munroe et al., 2010); rating of current risk of danger from the other parent; and measures of interparental conflict (2) Criminal, Traffic, and Civil Court and Public Records indicating DFV	(1) No sig. diff across the study conditions on the MASIC scores; rating of current risk of danger from the other parent; or other measures of interparental conflict at 1 year follow-up (2) No sig. diff across study conditions on measures related to court records indicating DFV at 1 year follow-up
Holtzworth-Munroe, Beck, et al. (2021), USA	To compare traditional litigation with two mediation approaches designed to protect parent safety-shuttle and videoconferencing.	Quantitative (RCT, cross-sectional)	Parents (*n* = 196 cases; *M*_age_ = 32.1) seeking to resolve their separation- or divorce-related disputes in Washington, DC and reporting high levels of DFV; traditional litigation (*n* = 67); shuttle mediation (*n* = 64); videoconferencing mediation (*n* = 65); 86% Black, 6% Hispanic; *M*_edu_ = 13.4 years; 79% employed; income $38,921	Case management pathway (traditional litigation, shuttle mediation, videoconferencing mediation)	Subjective report via Parent Immediate Outcome Form—a questionnaire designed for the study which gathered information about safety and satisfaction with proceedings	On a composite scale of five-items measuring parents’ feelings of safety or fear during the litigation process, relative to parents in the traditional litigation condition (*M* = 5.28, *SD* = 1.63), parents in mediation (*M* = 6.16, *SD* = 1.13) reported feeling safer and less fearful, *z* = 3.00; *p* < .007. Parents in shuttle and videoconferencing mediation did not differ significantly
Jiang et al. (2023), USA	To explore whether reaching an agreement in mediation designed to protect parent safety impacts party and family outcomes for parents reporting high levels of DFV	Quantitative	Parents (*n* = 165 cases; *M*_age_ = 32.6) seeking to resolve their separation- or divorce-related disputes and reporting high levels of DFV; *M*_edu_ = 13.5 years; 79% employed; income $39,131	Whether agreement was reached in mediation; whether agreement was not reached and the case returned to court; whether the case went to court without attempting mediation	(1) IPV composite variable (α = .74): A new variable that summed the *z*-scores of four measures from Holtzworth-Munroe, Beck, et al. (2021). Three were derived from the MASIC IPV measure (Holtzworth-Munroe et al., 2010). The fourth measure, Physical Danger (from Supervised Visitation and Exchange Program Brief Safety Measure; Saunders et al., 2005), averaged two items (e.g., “Overall, please rate how high you think your current risk of physical danger is”) (2) Five-item questionnaire related to feelings of safety	(1) No sig. diff on the IPV composite variable across study conditions. (2) At immediate outcome, the mediation agreement group (*M* = 6.35, *SD* = 1.07 [mother]; *M* = 6.43, *SD* = 0.84 [father]) reported feeling safer than the no mediation agreement (*M* = 5.87, *SD* = 1.08 [mother]; *M* = 6.20, *SD* = 1.29 [father]), *t* = −2.23, *p* = .03*. Additionally, the mediation group reported feeling safer than the court group (*M* = 4.92, *SD* = 1.50 [mother]; *M* = 5.65, *SD* = 1.69 [father]), *t* = −3.52, *p* < .001***
Laing (2016), Australia	To explore the experiences of DFV survivors attempting to navigate safe post-separation parenting arrangements through the Australian family law system	Qualitative (Interviews)	Women (*N* = 22, aged 24–54) recruited through flyers distributed by five DFV services; 23% CALD background, SES varied	NA	Subjective report of safety via interview	Women provided examples of effective strategies to protect themselves and their children from post-separation abuse, for example, seeking specialist DFV services that provided validation, practical support, information, and referrals to supportive lawyers; and proposing contact arrangements that minimized children’s exposure to further abuse
Lapierre et al. (2022), Canada	To investigate children’s relationships with their fathers (or mother’s partners) in the context of DFV, before and after separation	Qualitative (Interviews)	Children (*N* = 59; 61%; *M*_age_ = 11.0) recruited through DFV shelters, community organizations, and child protection agencies. 61% from Quebec, 39% from Ontario; all identified as Francophone	N/A	Subjective report via interview of safety and wellbeing	Limited contact in the post-separation period was seen by most children as a positive outcome regarding their safety and wellbeing. Most (percentage NR) children felt relief to be safe and reported less hypervigilance in their attempts to protect themselves and their mothers
Meyer (2014), Australia	To examine DFV survivors’ short- and long-term experiences of safety and wellbeing after being supported through a 6-week police-led integrated response to DFV	Mixed methods (Surveys and interviews) T1 (Initial), T2 (6 weeks), T3 (3 months-interview only)	Women (*N* = 78) considered high-risk and engaged with an integrated DFV response (*n* = 7 interviewed at T3); all had children	Integrated police-led intervention involving three key partner agencies (probation, child safety, DFV service) sharing information, supporting referrals, monitoring children’s safety, and perpetrator compliance with IVOs	Survey (eight-item questionnaire capturing women’s self-rated level of safety and wellbeing at initial contact and 6 weeks post-intervention) and subjective report via interview	*Survey*: Women reported significantly increased physical safety for themselves (T1 *M* = 3.35, T2 *M* *=* 4.64 [*SD*: NR], *p* < .01) and their children (T1 *M* = 3.84, T2 *M* = 4.75 [*SD* NR], *p* < .01) immediately post-intervention *Interview*: Women reported improved safety immediately after the initial separation from an abusive partner (e.g., due to access to crisis accommodation) however identified that ongoing support was essential to ensure that initial improvements in safety were sustainable over time, for example, to establish housing/financial stability, social support, and sense of identity
Nikupeteri et al. (2023), Finland	To analyze children’s experiences of post-separation stalking as a form of DFV, and how the Capabilities Approach can assist social workers to understand the issue and support children	Qualitative (Interviews)	Children and young people (*N* = 18; 83%; aged 4–21) whose father or stepfather had stalked their mothers after separation, recruited from the national Stalking Support Centre in Finland	N/A	Subjective report via interview of feeling safer	Children gained a sense of safety after supervised or suspended contact with their father; younger children emphasized concrete, physical devices such as locks and alarm systems that contributed to enhanced feelings of safety
Shalansky et al. (1999), Canada	To understand the experiences of abused women who, through court-imposed custody and access orders, continue to be involved with abusive ex-partners; and to describe common factors which affect the experiences of these women	Qualitative (Interviews)	Women (*N* = 5, aged 35–45) recruited from follow-up programs at a Canadian transition house for abused women; all had children (*M*_age_ = 9.5)	N/A	Subjective report via interview of feeling safer	Essential factors associated with increased safety (self-reported) included interaction with formal systems of support (police, legal, healthcare, housing referral services), and the quality of professional knowledge and understanding within these systems about abuse and risk. Distancing from their abuser was key to perceived safety, although often contradicted or complicated by the perpetrator’s legal right to custody of/contact with their children
Zeoli et al. (2013), USA	To examine women’s responses to DFV committed by ex-husbands with whom they had undergone custody disputes	Qualitative (Interviews)	Women (*N* = 19, *M*_age_ = 40) who had divorced DFV-perpetrating husbands, located through publicly available family court divorce records; 90% white, 5% Black, 5% Latina; all had children (aged 3–25)	N/A	Subjective report via interview of feeling safer	Women reported effective strategies to protect themselves and their children including setting boundaries to govern their interactions with ex-husbands, for example, not being present during child exchanges. The justice system was perceived to provide greater safety and support (e.g., IVOs) than the family court. The latter was felt to be particularly unhelpful in providing protection for their children

*Note.* All ages reported in years; average income = average annual salary of the sample in USD; N/A = not applicable; NR = not reported; DFV = domestic and family violence; FL-DOORS = family law detection of overall risk screening; TFCC = technology-facilitated coercive control; SFT = speaking for themselves; MASIC = mediator’s assessment of safety issues and concerns; IPV = intimate partner violence; CALD = culturally and linguistically diverse; SES = socioeconomic status; IVO = intervention order.

* Remainder of sample were in a relationship and excluded.

** Only qualitative component relevant and extracted.

*** No gender split reported for the finding.

### Synthesis of Results

To reflect our interest in individual and systemic factors associated with improved safety outcomes, a bioecological systems perspective (Bronfenbrenner & Morris, 2007) was used as a framework to guide analysis and synthesis of the included articles. Accordingly, we extracted data relevant to microsystemic (e.g., family/friends), and exosystemic (e.g., community services) levels, noting embedded individual behaviors within both. Synthesis involved becoming familiar with the data, grouping factors according to the corresponding systemic level, and reporting the analysis in the results section.

## Results

### Selection and Characteristics of Sources of Evidence

**Figure 1.**
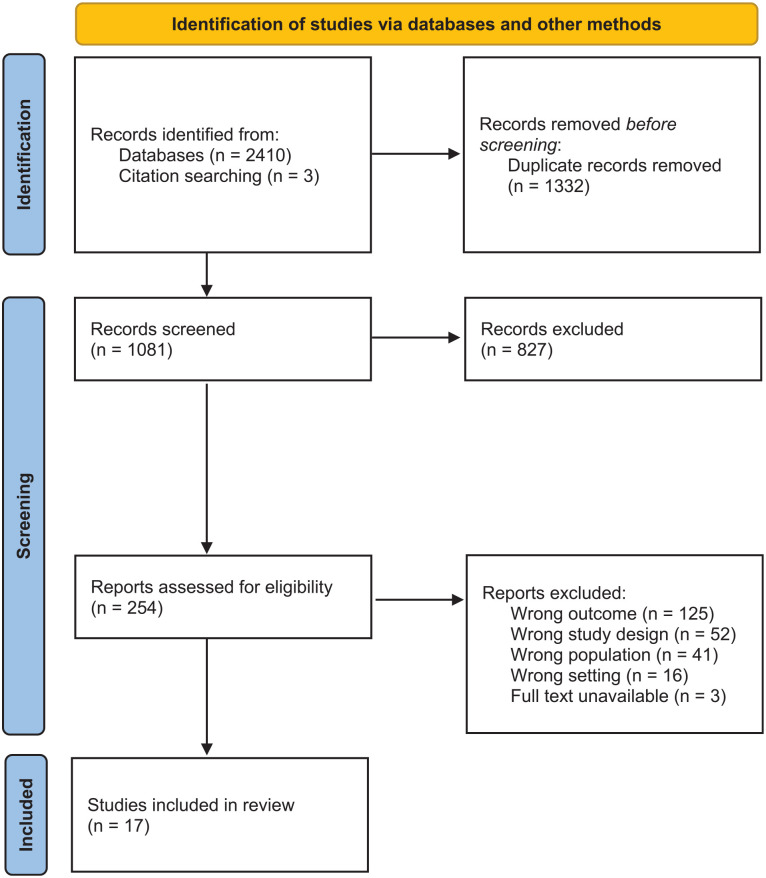
PRISMA diagram.

In total, 17 studies met the inclusion criteria. Results are summarized in Figure 1 via a Preferred Reporting Items for Systematic Reviews and Meta-Analyses’ (PRISMA) diagram.

Of these 17 studies, seven were from the United States, four from Australia, three from Canada, and one each from Sweden, Norway, and Finland. The research spanned over two decades, from 1999 to 2023. Eleven studies used qualitative methods (primarily interviews), four were quantitative (of which two were longitudinal), and two were mixed methods. Study sample sizes ranged from 5 to 196. Three studies included overlapping samples (Holtzworth-Munroe, Applegate et al., 2021; Holtzworth-Munroe, Beck, et al., 2021; Jiang at al., 2023). Participants were recruited from a variety of settings including adults and children who were accessing DFV shelters and services or involved in the family law and/or criminal justice system. Unless sampled from that specific setting, involvement in the family law system was not explicit. Three studies included the direct involvement of children and young people (two involving children only) reporting on their own safety; 10 studies involved women (2 reporting on their own safety, the remaining 8 also reporting on their child’s safety); and 5 involved both women and men (4 reporting on their own and their child’s safety; 1 reporting on their child’s safety only). Most articles did not report how long the parties had been separated. Of those that did report, length of separation varied from 6 months to 8 years. See Table 2 for further characteristics of included studies.

### Results of Individual Sources of Evidence

See Table 2 for a detailed summary of findings.

### Measurement of Safety and Study Design

The way that safety was measured varied across studies. For nine studies (Boethius et al., 2023; Clough et al., 2013; Dragiewicz et al., 2021; Hauge & Kiamanesh, 2020; Laing, 2016; Lapierre et al., 2022; Nikupeteri at al., 2023; Shalansky et al., 1999; Zeoli et al., 2013), safety was not explicitly measured as a primary outcome and emerged as a secondary finding. These were qualitative studies examining survivors’ experiences of DFV more broadly and/or their interactions with relevant supports and services.

For those that included a measure, five studies (Booth et al., 2023; Holtzworth-Munroe, Applegate, et al., 2021; Holtzworth-Munroe, Beck, et al., 2021; Jiang at al., 2023) utilized a subjective report of increased safety (e.g., self-report questionnaire indicating increased safety). Four studies (Bomsta & Sullivan, 2018; Fotheringham et al., 2013; Fox et al., 2018; Meyer, 2014) were specifically focused on safety following intervention. Of these, two measured safety at follow-up via interview (Bomsta & Sullivan, 2018; Fotheringham, 2013), one used scaled survey data (Fox et al., 2018), and one utilized both an interview and survey (Meyer, 2024). Two studies incorporated a file analysis component (Fotheringham, 2013; Holtzworth-Munroe, Applegate, et al., 2021). Overall, studies that involved qualitative methods yielded critical information beyond that gained from the quantitative studies, by elaborating on the circumstances in which relevant factors contributed to reports of felt safety from the perspective of the survivor.

### Synthesis of Results

Data from the included studies were organized into two main categories, informed by a bioecological systems perspective (Bronfenbrenner & Morris, 2007). In line with the *microsystemic* level of this framework, safety factors emerged within the individual’s immediate environment and informal relationships. At the *exosystemic* level, safety factors were related to certain aspects of services and the family law system. The dominant theme emerging from this synthesis of findings was the active role that the survivor had in creating and navigating safety for themselves and their children. We elaborate on this theme below, disaggregating the nature of interaction with their immediate environment, relationships and services, and processes within the wider service system that proved effective in enhancing safety outcomes.

#### Microsystemic Factors

##### Physical Distance

A number of studies demonstrated that physical separation (Meyer, 2014; Shalansky et al., 1999), including the active creation and maintenance of distance (Nikupeteri et al., 2023) and physical safety devices (e.g., locks and alarm systems; Nikupeteri et al., 2023), were effective in providing immediate safety for caregivers and children. At times, physical separation was made possible with the assistance of informal supports (e.g., friends or family; Clough et al., 2014).

##### Strategic Use of Technology

Women further implemented tactics such as the use of technology to increase their safety. This included finding support and information (e.g., through online resources and groups), storing and recording evidence, keeping in touch with support networks (Boethius et al., 2023), or avoiding escalation in risk by purposely abstaining from technology use or enduring electronic surveillance (Dragiewicz et al., 2021). The latter was found to be particularly helpful in the absence of adequate social, legal, and structural responses to violence (Dragiewicz et al., 2021).

##### Relational Support

In addition to tapping into relational resources themselves, caregivers enhanced their children’s safety directly through the provision of relational support. Key tactics included normalizing new living arrangements and maintaining predictable routines, in order to protect them and establish a sense of stability (Hauge & Kiamanesh, 2020). Familiarity and predictability were considered prerequisites for children to experience a sense of safety and control in new environments, particularly after periods of turmoil and upheaval (Hauge & Kiamanesh, 2020). Of note, such personal and relational responses were contextual, likely embedded within and enabled by legal protection, financial assistance, and social support (Bomsta & Sullivan, 2018; Hauge & Kiamanesh, 2020; Shalansky et al., 1999).

#### Exosystemic Factors

##### Responsiveness and Adequacy of Services

Multiple characteristics of formal services (e.g., police, legal, healthcare, DFV services) were associated with safety for families. At the practitioner level, this included the presence of well-trained staff (Clough et al., 2014; Shalansky et al., 1999) who were responsive (Zeoli et al., 2013), actively advocated (Clough et al., 2014), provided practical information and support (e.g., flexible funding, housing/temporary accommodation; Bomsta & Sullivan, 2018; Hauge & Kiamanesh, 2020; Meyer, 2014), were validating, and made appropriate referrals (e.g., to supportive lawyers; Laing, 2016). However, the agency of women remained key to navigating their safety in the face of barriers when accessing such services (e.g., staying with friends if there were no appropriate public housing properties available; Clough et al., 2014) or collecting their own evidence if police failed to investigate their reports (Dragiewicz et al., 2021).

The critical importance of service responsiveness to the unique needs of each family and collaborative practice including information sharing with other services was a central finding (Fox et al., 2018; Meyer, 2014). The longer term and flexible nature of support were significant (Bomsta & Sullivan, 2018; Fox et al., 2018), and stable housing was a key factor (Bomsta & Sullivan, 2018; Fox et al., 2018) to retain the immediate gains that families made from service support (Meyer, 2014).

##### Processes and Outcomes in the Family Law System

There were notable findings for studies that explicitly identified involvement with the family law system. Firstly, the process of screening for DFV proved effective in itself. Booth et al. (2023) found that this led to increased safety for parents in a mediation context. Certain processes within the family law system enhanced felt security. For example, parents who went through shuttle and videoconferencing mediation felt safer than those allocated to traditional litigation (Holtzworth-Munroe, Beck, et al., 2021). While this observed increase for those in mediation did not hold after 1 year, safety did not decrease (i.e., parents neither suffered nor showed benefits compared to cases in traditional litigation), indicating the appropriateness of mediation as a safe alternative to court in certain circumstances; Holtzworth-Munroe, Applegate, et al., 2021).

Additionally, certain outcomes were associated with feelings of safety. For example, caregivers whose mediations resulted in full or partial agreement reported feeling safer than those who did not agree, or those whose matter was heard in court without attempting mediation (Jiang et al., 2023). Supervised or suspended contact between children and the abusive parent was associated with enhanced safety of the survivor parent (Laing, 2016; Lapierre et al., 2022; Nikupeteri et al., 2023)—and notably, from the perspective of children (Lapierre et al., 2022; Nikupeteri et al., 2023).

Children expressed feeling safer through court interactions and processes that supported their safety and allowed a safe space to express themselves (Fotheringham et al., 2013). This included integrative responses where lawyers worked alongside therapists, leading to legal decisions that positively impacted their safety (Fotheringham et al., 2013). If contact between children and the abusive parent was ordered, the protective parent reported actively strategizing to create safety from post-separation abuse (e.g., by not being present during child exchanges or utilizing the justice system for support; Zeoli et al., 2013).

## Discussion

### Summary of Evidence

In seeking to identify modifiable factors associated with increased safety for separating families affected by DFV, we first identified four limitations in the existing body of literature: paucity of research focusing specifically on safety as a primary outcome, inconsistency in definition or measurement where it is included, lack of clarity regarding active involvement with the family law system, and methods containing little intentional focus on what works to increase safety for this highly vulnerable group.

In the 17 studies examined, we identified an array of interacting multi-systemic factors associated with growing safety in the face of DFV risk. Safety emerged in the movement from physical separation to the survivor’s use of resources, including relational support and strategic use of technology, together with responsive, tailored assistance of skilled practitioners and services with a survivor-centered approach. When active systemic barriers and service access limitations arose, the survivors’ own initiative appeared to be linked with enhancing their sense of safety, contingent on the availability of resources (e.g., social networks to provide shelter, financial means to access technology). Research has demonstrated variable success for survivors left strategizing without adequate intervention and support (Vikander et al., 2023). Thus, consistent efforts to improve responses within systems and among providers is key. The intentional protective use of technology expands Bronfenbrenner’s concept of the microsystem to the *virtual* microsystem (Navarro & Tudge, 2023). Ultimately, our findings underscore the context-dependent nature of safety and the complex interactional process between the survivor, resources, supports, and systems. See Table 3 for a summary of critical findings.

**Table 3.** Critical Findings.

- Research focusing specifically on safety as a primary outcome for separating families affected by DFV is scarce - When safety is included, it is inconsistently defined and measured - Methods contain little intentional focus on what works to enhance it - Involvement with the family law system is often not explicit - Findings indicate interacting multi-systemic factors associated with enhanced safety, including: geographic distance, protective use of technology, relational support, the assistance of survivor-centered practitioners and services, and certain aspects/outcomes of the family law system (i.e., early risk screening, specialized mediation, and supervised or suspended contact) - In the face of systemic barriers or service limitations, caregivers are compelled to actively navigate safety for themselves and their children, demonstrating the need for enhanced service responses and resources

*Note*. DFV = domestic and family violence.

The review identifies key family law processes that offered ongoing protection for separating families, beyond informal or formal supports and services. Most recently we see evidence for specialized mediation designed to protect parent safety (e.g., videoconferencing or shuttle mediation, facilitated by mediators specifically trained in DFV) compared to traditional litigation (Holtzworth-Munroe, Beck, et al., 2021) and participation in universal risk screening (Booth et al., 2023) as part of the safety-enhancing environment. Again, when services in this context did not in themselves provide long-term safety, families were often required to independently strategize to increase safety (e.g., by setting boundaries to limit unsafe interactions with former partners; Zeoli et al., 2013). To encourage safer outcomes, prior research has indicated the importance of expanded access to affordable legal representation for survivors to be appropriately heard by key decision-makers; in-depth training for court professionals (e.g., judges, family report writers, mediators, lawyers) to ensure accurate identification and responses to DFV (particularly coercive control and legal abuse); and safe child contact procedures or facilities for supervised contact and exchanges (Gutowski & Goodman, 2020).

Our review highlights a shift toward qualitative methods as an important step in DFV research to give voice to those most significantly impacted by the issue. Such methods were able to provide insight to survivors’ experiences of safety, along with socioecological factors associated with feelings of safety. In the court context, for example, both caregivers and children reported that supervised or suspended contact between children and the abusive parent was associated with subjective feelings of increased safety (Laing, 2016; Lapierre et al., 2022; Nikupeteri et al., 2023). Given DFV research is generally limited in its risk-oriented approach, the intentional examination of safety from the perspective of children and caregivers, utilizing qualitative methods, is essential.

### Implications

Physical separation and cessation of contact may be considered a fundamental prerequisite to recovery and healing for caregivers and children affected by DFV (Smith, 2023). It may allow the space to establish a sense of safety and security that extends beyond a physical sense (e.g., toward financial, emotional, and psychological safety/security). While recovery is not a linear process, it is difficult to contemplate how the journey can begin if caregivers and children are to have ongoing contact with their abuser through court-ordered processes and conditions, if safety has not yet been established and the restoration of an adequate sense of trust has not been achieved (Herman, 2015). Overall, there remains a call to action for increased recognition that survivors’ need for intervention, and protection does not end at relationship separation, but in fact increases (Anderson & Saunders, 2003). Table 4 summarizes key practice, policy, and research implications arising from this scoping review that appear central to enhanced safety for caregivers and children.

**Table 4.** Implications for Practice, Policy, and Research.

*Practice implications* - Focus on early and accurate identification of DFV and ensure that subsequent safety responses are collaborative and survivor-centered, while holding the perpetrator to account - Training and education in services and systems that engage with survivors to increase knowledge and understanding of DFV *Policy implications* - Target funding toward evidence-based, survivor-informed initiatives seeking to enhance safety, for example, flexible funding, accessible housing, long-term support structures, specialized mediation, early risk screening - Utilize survivor-centered instruments when developing and evaluating policy changes intended to improve safety, including the voice of the child *Research implications* - Further research required in the family law context that intentionally sets out to examine safety, defined and measured in ways that resonate with the lived experience of child and adult survivors; incorporates longitudinal designs, to examine safety over time; recognizes intersectionality of survivors; and better tracks demographic data to understand nuances related to their safety needs

*Note*. DFV = domestic and family violence.

Findings highlight the importance of accurate and early identification of DFV during relationship separation, with particular emphasis on collaborative and trauma-informed responses in this context. Alongside existing efforts to hold perpetrators to account (e.g., First Step, 2023), our findings demonstrate the importance of supporting the fundamental needs of survivors. This includes viewing and responding to safety needs from their perspective, as the experts of their situation, enabling their capacity to engage in self-protecting actions, and ensuring that supports and services are equipped to respond.

Additionally, research translation is needed to ensure mutual, coordinated, and intentional processes in practice: processes that are based on shared responsibility between families and the broader system, and are underpinned by legislative support, and practice direction. Notably, some countries are making progress on this front. A promising approach in Australia is the recent introduction of Lighthouse—an innovative approach to the screening and management of risk, which allows the Federal Circuit and Family Court of Australia to shape the allocation of resources and urgency to cases involving DFV and other safety risks (Australian Law Reform Commission, 2019). Disclosures during the screening process are confidential and inadmissible as evidence. As we can see from historical reforms, however, well-intended changes do not necessarily translate to safer outcomes for families (Kaspiew et al., 2010). While promising, the continued evaluation of these changes is essential to ensure that they translate to the intended safety outcomes for families. The voice and perspective of the survivor is crucial in such research.

### Limitations

The limitations of this review provide an important context for interpretation of our findings. In examining the scope of literature focused on safety, there were a number of methodological issues influencing the available information. Conceptualization and measurement of two key concepts within this review, *safety* and *separation*, were nuanced and varied in the literature. Thus, while efforts were made to carefully broaden these concepts within the search strategy and to locate papers using snowballing methods, it is possible that papers relevant to this review were not captured. Further, it is possible that included studies reported on findings where participants reflected on strategies used prior to relationship separation—despite being separated at the time of the study. This may be overcome in future studies by purposefully recruiting participants in the active phase of separation, where they are physically separated and involved with the family law system. Diversity was minimally discussed in the studies reviewed. The experience of safety is impacted by intersectionality—socioeconomic status, cultural background, ability, sexual orientation, gender identity, immigration status, and other variables (A. S. Day & Gill, 2020)—and generally, most studies did not include a detailed report of demographics nor consider their impact. Future research should acknowledge the safety needs and experiences of diverse groups, by paying particular attention to marginalized groups that are disproportionately affected by DFV and face additional barriers to accessing relevant supports and services (Tayton et al., 2014). For example, in Australia, Indigenous people are 32 times more likely to be hospitalized for DFV than their non-Indigenous counterparts (Australian Institute of Health & Welfare, 2019) and may experience various barriers to support due to historical trauma, cultural disconnect, and ongoing systemic inequalities.

While the aim of this review was to identify and map the existing evidence on this topic, the scoping methodology did not account for an assessment of the reliability or validity of the findings from eligible studies. Hence, the conclusions drawn from this review may be less robust than a systematic methodology.

Finally, examining safety factors associated with specific types of abuse was beyond the scope of this review. Future research is invited to explore safety enhancement for children in different contexts (e.g., exposure to DFV compared to direct experiences of abuse). While these issues are shown to overlap (Callaghan et al., 2018), there may be differences worthy of research attention.

### Future Research

The dearth of evidence uncovered by this review invites future research to further determine factors associated with safety for this vulnerable population. Translation of findings by various stakeholders (e.g., legal systems, DFV services) into safety-enhancing interventions and processes is essential. Importantly, for the system to be more responsive to the lived experience and safety needs of survivors, future research would optimally facilitate opportunity to safely express their views and concerns. This includes going beyond objective, quantitative measures of reported risk/victimization by learning what safety means to child and adult survivors, how it feels, and *what works* to increase their safety during separation and in the longer term. Hence, their voice is essential in not only the shaping of responses but also in the evaluation of interventions and processes seeking to enhance their safety (Mengo et al., 2023).

While the role of parental safety and well-being in children’s safety cannot be understated (Francia et al., 2020), children require recognition as victim survivors in their own right. Their voices provide valuable insight into factors and conditions that enhance their safety (Fotheringham et al., 2013; Lapierre et al., 2022; Nikupeteri et al., 2023); however, such perspectives are seemingly absent from the literature. The inclusion and centrality of children in future research is vital, particularly in light of established evidence of the pervasive harms of DFV (Abordo et al., 2023). Inclusion of the voice of child and adult survivors is in line with one of the key messages from the Victorian Royal Commission into Family Violence—the need to embed the lived experiences of victim survivors in all aspects of the family violence system and responses to family violence (Domestic Violence Victoria, 2020). It is noted that children’s safety is paramount in research and family law processes incorporating their perspective. Careful consideration and planning are vital to ensure that they are not placed at increased risk of harm or re-traumatization (Morris et al., 2012).

## Conclusions

Examining evidence for factors associated with safety in separating families affected by DFV is key to developing a responsive system. Our findings show that long-term safety outcomes require appropriate responses from formal services and supports, guided by the specific needs of caregivers and their children. The family law system is a key context impacting safety. Our findings suggest that parties are compelled within this context to actively strategize to achieve safety, likely due to barriers such as misidentification or under detection of DFV, and lack of appropriate and coordinated responses when risk is disclosed. The intersecting role of broader cultural and societal forces on safety in the face of DFV is clear.

Importantly, this review highlights a need for better articulation of what safety means for survivors, and how it may be conceptualized as a shared goal in practice. It emphasizes the importance of survivor-centered contributions to the development and evaluation of safety interventions, to ensure that they are working as intended. The predominant risk-oriented focus of current research is limiting, diverting attention from the experience of the survivor and from the centrality of forming a shared path toward safety. On top of that, a focus on enhanced safety may render the experience of children more visible. Above all, this review suggests that safety enhancement is not the opposite of risk management, and that survivor safety represents something beyond the risk of victimization.

## Appendix

*Screening Tool*

1. Does the *title or abstract* use English?
a. Yes: continue screening
b. No: stop screening
2. Does the *title or abstract* indicate that a review was conducted?
a. Yes: tag as “review” and stop screening
b. No: continue screening
3. Does the *title or abstract* indicate that this is an empirical study?
a. Yes or Unsure/Unclear: continue screening
b. No: stop screening

*Abstract Screening*

4. Does the *abstract* indicate that a sample of caregivers/children were studied?
a. Yes or Unsure/Unclear: continue screening
b. No: stop screening
5. Does the *abstract* indicate that family violence was studied?
a. Yes or Unsure/Unclear: continue screening
- Key words: Family violence, domestic violence, intimate partner violence, domestic abuse, partner abuse, spousal abuse
b. No: stop screening
- Key point: “high conflict” or “abuse” in the absence of any related “family violence” terms above are NOT eligible
6. Does the *abstract* indicate that family violence was studied in the context of separation?
a. Yes or Unsure/Unclear: continue screening
- Key words: Family court, law system, legal system, family law, separation, divorce, mediation, dispute resolution
b. No: stop screening
- Key terms: intact families
7. Does the *abstract* indicate that the study focuses on increased safety as a primary outcome?
a. Yes or Unsure/Unclear: continue screening
- Key words: safety, security, diminished risk, decreased risk, decreased victimization, protection
b. No: stop screening
- Key point: if the study focuses on factors associated with increased risk, tag as “increased risk” and stop screening; if the study focuses on outcomes that imply safety but do not articulate a clear measure/outcome of safety, tag as “important but irrelevant outcome” and stop screening

* Decision: Should this article be included?*

a. *Yes*, all screening questions answered Yes or Unclear
b. *No*, at least one answers definitely “No”
